# Engineering physical microenvironments to study innate immune cell biophysics

**DOI:** 10.1063/5.0098578

**Published:** 2022-09-20

**Authors:** Nikita Kalashnikov, Christopher Moraes

**Affiliations:** 1 Department of Chemical Engineering, McGill University, Montreal, Quebec H3A 0G4, Canada; 2 Department of Biological and Biomedical Engineering, McGill University, Montreal, Quebec H3A 0G4, Canada; 3 Goodman Cancer Research Center, McGill University, Montreal, Quebec H3A 0G4, Canada; 4 Division of Experimental Medicine, McGill University, Montreal, Quebec H3A 0G4, Canada

## Abstract

Innate immunity forms the core of the human body's defense system against infection, injury, and foreign objects. It aims to maintain homeostasis by promoting inflammation and then initiating tissue repair, but it can also lead to disease when dysregulated. Although innate immune cells respond to their physical microenvironment and carry out intrinsically mechanical actions such as migration and phagocytosis, we still do not have a complete biophysical description of innate immunity. Here, we review how engineering tools can be used to study innate immune cell biophysics. We first provide an overview of innate immunity from a biophysical perspective, review the biophysical factors that affect the innate immune system, and then explore innate immune cell biophysics in the context of migration, phagocytosis, and phenotype polarization. Throughout the review, we highlight how physical microenvironments can be designed to probe the innate immune system, discuss how biophysical insight gained from these studies can be used to generate a more comprehensive description of innate immunity, and briefly comment on how this insight could be used to develop mechanical immune biomarkers and immunomodulatory therapies.

## INTRODUCTION

Cells of the innate immune system, notably neutrophils and macrophages, circulate within blood vessels or lay dormant in various tissues until the body mobilizes them to deal with infection,^1^ injury,^2^ and/or foreign objects.^3,4^ After reaching the affected tissue,^5^ these cells use the various tools at their disposal to control and resolve the threat, initially promoting inflammation and later re-establishing favorable conditions for tissue homeostasis.^6,7^ In contrast, when the threat is insurmountable, excessive innate immune activity can be damaging, causing loss of tissue function through fibrosis^8,9^ or chronic inflammation, and can drive diseases such as atherosclerotic heart disease, chronic obstructive pulmonary disease, and arthritis.^10–12^

Most differentiated cells are highly responsive to their surroundings, particularly elements of the biophysical microenvironment. These mechanical factors are now recognized as being pivotally important in driving cell fate and function.^13^ However, innate immune cells are not always committed to one location in the body and it would, therefore, be reasonable to assume that they do not have the same dependence on environmental biophysics. Several recent observations made with implanted biomaterials suggest otherwise: an optimal size of 1.5 mm for spherical implants results in less leukocyte recruitment,^14^ parallel uniaxial topography with characteristic lengths similar to the cellular length scale promotes macrophage polarization into an anti-inflammatory phenotype,^15^ while excessively stiff hydrogel implants chronically recruit neutrophils and result in a loss of anti-inflammatory macrophage markers.^16^ In contrast, unaltered tissue-derived extracellular matrix (ECM) scaffolds do not develop fibrous capsules after implantation,^17,18^ demonstrating why it is critically important to consider these physical parameters in designing biomaterials^19,20^ and regenerative therapies^21^ that interact with the immune system. Despite these developments, we still do not have a complete understanding of innate immunity from a biophysical or mechanical perspective, especially considering that core neutrophil and macrophage functions such as migration^22–24^ and phagocytosis^25,26^ are intrinsically mechanical processes.

Ever since the discovery of the molecular components of the cytoskeleton and the various mechanotransductory pathways that direct gene expression and cell fate,^27–30^ engineers have been motivated to develop tools to measure cell-generated mechanical forces and to build microenvironments to explore how external physical cues drive cell behavior.^31–34^ Over the last 20 years, a large focus of cell biomechanics^35,36^ and mechanobiology^37,38^ research has been on anchorage-dependent cells with far fewer studies focused on cells of the hematopoietic lineage despite their importance in homeostasis, development, and disease. This disparity can largely be attributed to the lack of engineered research platforms designed for innate immune cells, which are small, highly adaptive to local context and needs, and often non-adherent as well as too short-lived for cell culture unless immortalized. In this review, we aim to build on a recent appreciation for the physical nature of the immune system^39–43^ by surveying engineering tools that can be used to study the biophysics of innate immune cells as well as generate a comprehensive biophysical description of innate immunity. To do so, we highlight the biophysical events that characterize innate immunity, examine our current understanding of innate immune cell biophysics, and finally explore three key applications in migration, phagocytosis, and phenotype polarization.

## VIEWING INNATE IMMUNITY FROM A BIOPHYSICAL PERSPECTIVE

Among the various cells that make up the innate immune system, neutrophils and macrophages stand out the most: the former in their sheer quantity and the latter in their diversity. The body dedicates more than half of its red bone marrow to produce neutrophils, forming a large army that circulates in the blood after conception.^44^ In contrast, even though both neutrophils and monocyte-derived macrophages originate from the bone marrow in the adult, macrophages arise after a week in the embryo. First produced in the yolk sac and then in the fetal liver, they are seeded within various vital organs such as the brain, the lungs, and the heart, establishing various populations of tissue-resident macrophages that self-renew or are later replenished from circulating monocytes.^45,46^ These resident macrophages acquire specific functions associated with their local environment^47^ and are often responsible for recognizing threats and mounting initial innate immune responses.

### Reaching the threat

**FIG. 1.**
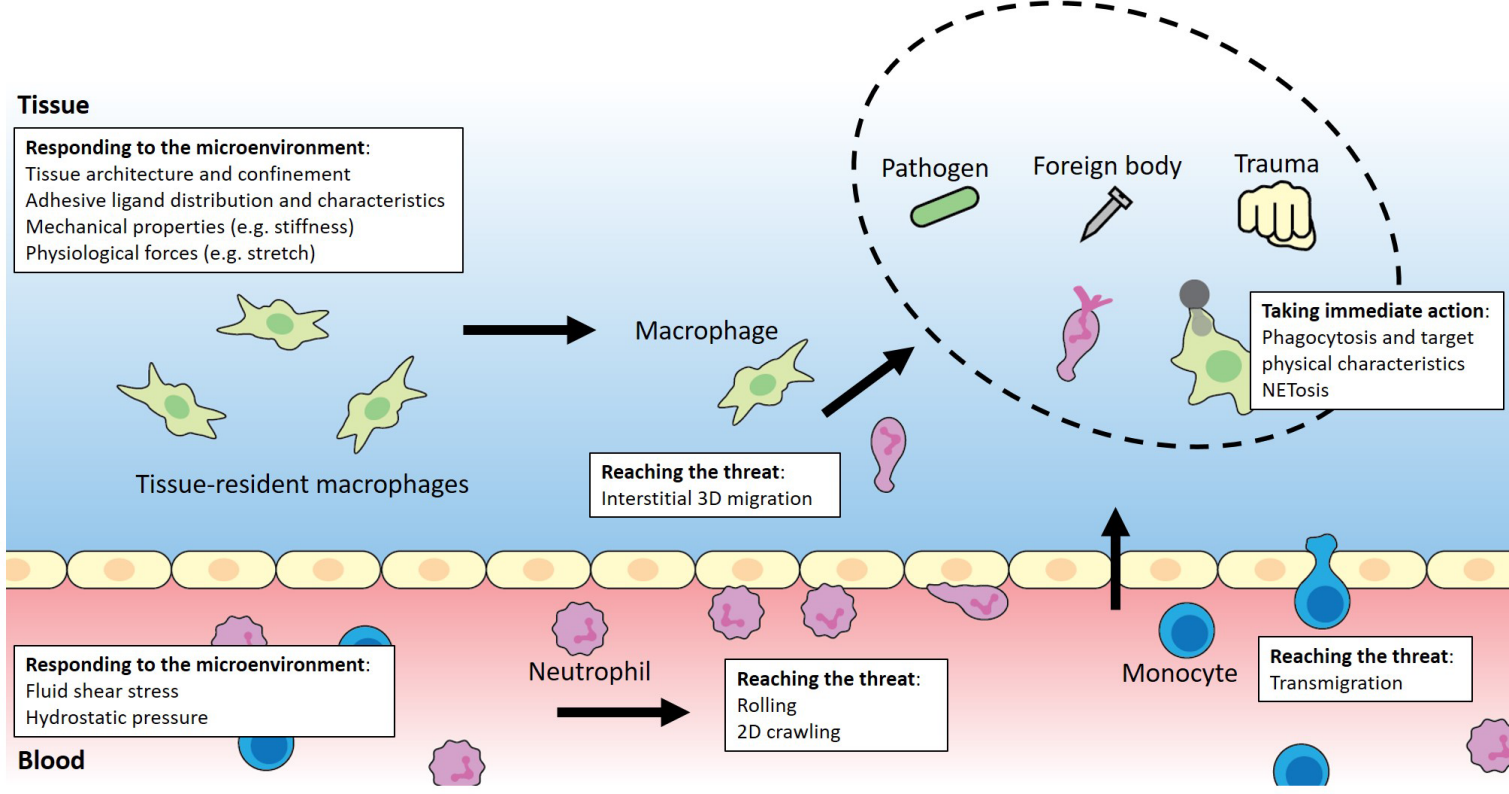
Biophysical journey of innate immune cells annotated to illustrate key physical events and various aspects of the physical microenvironment.

Irrespective of their origin, innate immune cells embark on a physically perilous journey across the body's various environments to reach an affected tissue and engage with a given threat (Fig. 1). Inflamed tissues and organs release potent chemical signals that drive neutrophils and monocytes out of the bone marrow or other reservoirs such as the spleen and the lungs and into the bloodstream to be rapidly transported to their destination. Along the way, these immune blood cells marginate closer to the endothelium, contact with it, and eventually form adhesions to it when they reach the low-flow postcapillary venule environment. Through initially transient and weak selectin-mediated adhesions, they begin to roll on the endothelial surface until they arrest and firmly attach to it by forming stronger integrin bonds in regions where activated endothelial cells display large amounts of chemokines on their luminal surface. Then, neutrophils and monocytes extend cellular protrusions and begin to crawl on top of the endothelial cells, prodding them to find a route through the endothelium into the underlying tissue.^48–51^ Inside of the affected tissue, neutrophils and monocytes – now differentiated into macrophages – joined by resident tissue macrophages physically migrate to reach the inflamed area by following concentration gradients of dissolved molecules (chemotaxis) or those bound to the ECM network (haptotaxis). Since rapid locomotion is essential for a proper efficient immune response, immune cells can migrate using an adhesion-independent ameboid mode that allows them to rapidly propel themselves through tissues. In addition, macrophages also have access to the slower adhesion-dependent mesenchymal migration mode that is common to adherent cells, which extend individual pseudopods to grasp their environment and then pull themselves forward. Using either of these modes, they migrate to finally reach their destination, whether it may be through loose fibrillar connective tissue and/or through the cell-packed parenchyma of an organ.^22,23,52^

### Taking immediate action

Although tissue-resident macrophages are typically the first to recognize pathogens, apoptotic cells, or foreign objects, all innate immune cells can detect threats at the affected site using cell–surface receptors for pathogen-, damage-, or lifestyle-associated molecular patterns (PAMPs, DAMPs, or LAMPs).^53^ Once detected, they can either initiate the mechanically intricate process of phagocytosis to engulf and digest the object or secrete strong chemical compounds such as reactive oxygen species (ROS) to break it down directly. Macrophages are often considered to be better phagocytes that can clear dead apoptotic or infected cells, while neutrophils to be better granulocytes that can secrete more enzymes. As a consequence, during inflammation and/or an infection, neutrophils are typically recruited to help eliminate the threat, but often die in the process to be then cleared by macrophages. Furthermore, neutrophils also often sacrifice themselves when they release their chromatin in the form of sticky neutrophil extracellular traps (NETs) to effectively capture and kill pathogens, creating pus in the process.^54,55^

### Responding to the microenvironment and coordinating long-lasting responses

Since the discovery of phagocytosis about a century ago, innate immune cells have been mostly treated as simple phagocytes; however, they possess a vast repertoire of regulatory cytokines that they can utilize not only to communicate amongst each other but also to direct the behavior of other cells such as fibroblasts and cells of the adaptive immune system.^56–59^ Furthermore, neutrophils and, in particular, macrophages exhibit exceptional plasticity and can adapt their phenotype in response to their microenvironment. As a result, innate immune cells can be reversibly activated and/or polarized to maintain a specific phenotype with a defined secretome that allows them not only to fulfill their trophic roles but also to orchestrate both desirable and undesirable tissue-wide responses such as acute and chronic inflammation, tissue repair and regeneration, adaptive immunity, fibrosis, and even tumorigenesis.^10,52,58,60–62^

Deriving from their primary role in host defense, macrophage activation has been traditionally categorized into two groups. A classically activated M1 macrophage is pro-inflammatory and displays powerful microbicidal activity, whereas an alternatively activated M2 macrophage is anti-inflammatory with immunoregulatory functions and/or wound-healing capabilities. Although macrophage polarization is not completely binary and should not be defined by two extremes,^57,63^ canonical *in vitro* chemical stimuli are primarily defined for these two states: M1 polarization is chemically induced with LPS and/or IFNγ, whereas M2 macrophages are generated with IL-4 and/or IL-10.^64^ As highlighted in the “Taking immediate action” subsection, the life of macrophages is intensely physical and it would be reasonably to expect their physical microenvironment to also influence their polarization state. After all, innate immune cells are exposed to varying physiological environments, ranging from pulsatile shear stresses in the pressurized vasculature to highly porous soft breathing lung tissue,^19,65^ and respond to external and internal physical trauma with inflammation.^2^ In addition, biologically driven disease progression leads to drastically altered tissue architectures and mechanical properties, which either affect the physical functioning of the tissue or further exacerbates its susceptibility to additional physical stimuli.^66,67^ For example, in atherosclerosis, macrophage polarization affects plaque stability and growth, which can have implications on luminal obstruction or plaque rupture and have fatal cardiac consequences.^68,69^ Furthermore, in the lungs, M2 macrophages often contribute to fibrotic pathology, which dramatically stiffens lung tissue and can impair breathing.^70^

Although there is currently no working model to understand neutrophil diversity due to challenges associated with their reduced diurnal lifespan and their inability to proliferate after terminal differentiation, circulating neutrophils do exhibit phenotypic changes as they age or mature such as nuclear hypersegmentation, enhanced integrin activation and increased capacity to form NETs. Furthermore, upon chemical stimulation, neutrophils can become activated or primed not only to spread and migrate more on surfaces but also to release more ROS and synthesize more cytokines. Specific activation states, as observed with macrophages, are yet to be elucidated in detail; however, the existence of tissue-specific neutrophils has been speculated and there is growing evidence that neutrophils can polarize into an anti-tumorigenic N1 or a pro-tumorigenic N2 state in the context of cancer depending on the surrounding cytokine environment.^10,40,58,71,72^

## IDENTIFYING BIOPHYSICAL FACTORS AFFECTING THE INNATE IMMUNE SYSTEM

### Mechanical properties of innate immune cells

Although genomics and proteomics have been successfully utilized to distinguish between various white blood cell populations, mechanical characterization of innate immune cells is appealing, because it holistically captures the global state of the cytoskeleton and can, as a result, dramatically simplify the interpretation of the innate immune phenotype. More specifically, differentiation of promyelocytic leukemia (HL60) cells into neutrophils leads to softening, but to stiffening for macrophages, as determined with contactless optical microfluidic stretching devices^73,74^ and later confirmed with a microplate squisher system for primary macrophages differentiated from monocytes.^75^ Furthermore, HL60-differentiated neutrophils are more elastic and solid-like, thereby better suited to respond to short timescale mechanical phenomena, whereas macrophages are fit for longer timescales, as they are more viscous and liquid-like. These mechanical adaptations appear to be specific and have functional roles since they allow neutrophils to transit through microfluidic constrictions faster and presumably facilitate macrophage migration in porous interstitial-like environments.^74^ These characteristics are inevitably linked to the cytoskeleton and a multitude of studies have demonstrated that actin,^74–78^ microtubule,^73^ and myosin dynamics^75,78^ all contribute to the final observable innate immune mechanical phenotype.

Aside from inherent differences in mechanical properties between the various innate immune cells, phagocytosis of various targets and inflammatory chemical stimulation differentially affect their mechanical properties. Although phagocytosis of crystal-like clofazimine leads to macrophage softening,^79^ phagocytosis generally leads to stiffening due to increased ROS generation, which inhibits ingestion of additional particles since deformability is a prerequisite for phagocytosis.^77,79,80^ As a result, there appears to be a limit to phagocytosis, which can either be met through the uptake of many small particles or a few large ones.^81^ Along with biophysical phagocytic stimuli, IFNγ and LPS cause both primary and cell line macrophages to stiffen,^75,77,82^ whereas TNFα/PGE2 leads to softening.^75^ Furthermore, potent fMLP neutrophil bacterial chemokine stimulation stiffens monocytes and neutrophils, but softens macrophages.^74,83,84^ Considering that macrophages also soften when seeded on more compliant substrates and/or when exposed to dynamic stretching,^77^ all of these observations suggest that innate immune mechanical characteristics are a common pathway for physical and chemical stimuli in determining cell function as has been previously demonstrated for adherent tissue cells.

### Attachment of innate immune cells to their microenvironment

**FIG. 2.**
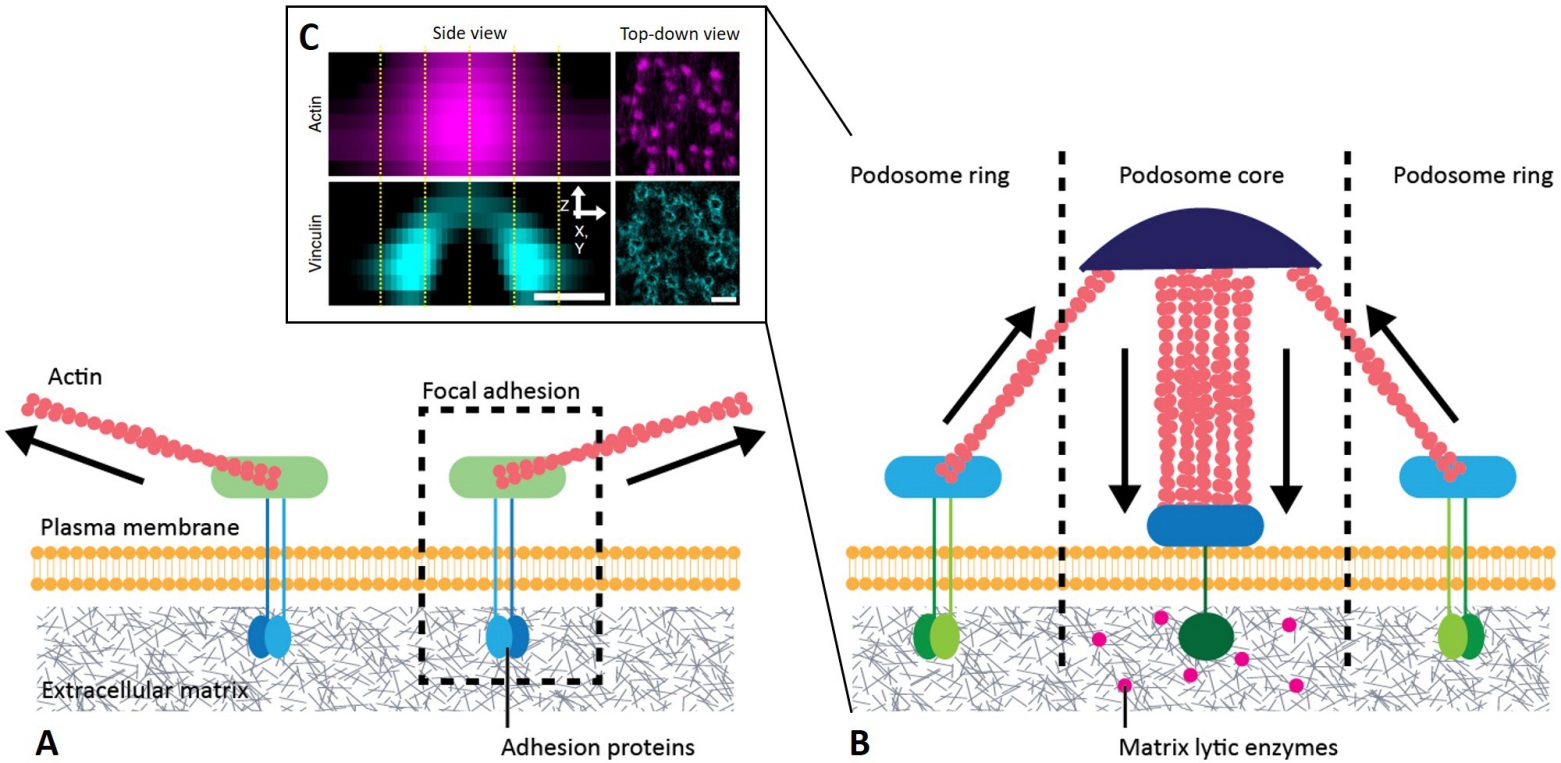
Cell–matrix attachments: (a) globally balanced pulling focal adhesions present in adherent tissue cells and (b) locally protrusive podosome unit characteristic of innate immune cells with (c) representative confocal images. [0.5 and 1 *μ*m scale bars; adapted from van den Dries *et al.*, Nat. Commun. **10**, 1–16 (2019).^105^ Copyright 2019, Authors licensed under a Creative Commons Attribution (CC BY) license.]

Most cells interact with their surroundings by forming cell–matrix adhesions through integrin–ligand binding events, which connect the internal cell cytoskeleton to external matrix molecules (Fig. 2). Epithelial and endothelial cells tightly attach to basement membranes with their intermediate filaments through hemidesmosomes,^85^ whereas most mesenchymal cells use their actin network to form focal adhesions [Fig. 2(a)], which they also exploit as signaling complexes to transmit and receive mechanical information.^86,87^ Although innate immune cells do not rely on adhesion for survival, neutrophils can attach and spread on and within scaffolds^76,88–90^ along with macrophages, which are inherently much more adhesive. In particular, macrophages predominantly attach to their environment through a smaller micrometer-sized type of actin-based cell–matrix adhesion complex, termed podosome, which is composed of an actin-rich core and a surrounding integrin adhesive ring^91–93^ [Fig. 2(b)]. Although they are made out of similar molecular components, focal adhesions are more stable and have actin filaments oriented tangentially to the cell membrane,^94^ whereas podosomes have a lifetime of a couple of minutes and have their actin filaments arranged perpendicular to the cell surface.^95^ Podosomes have been observed primarily in macrophages but have also been reported in neutrophils^96,97^ and hence may have an important role across the innate immune system.

### Podosome force generation and mechanical sensing functions

The specific architecture of cell–matrix adhesion complexes has profound implications on how cells generate forces and maintain a state of mechanical balance or tensional homeostasis with their surroundings. As in muscle, cells can use an actomyosin mechanism to contract and pull their surroundings toward them,^98,99^ allowing them to generate endogenous cytoskeletal tension to match the exogenous tension they sense from their environment.^86^ In order to maintain equilibrium, low motile anchorage-dependent cells must produce, at their focal adhesions, traction forces that balance each other out since these forces are applied in a direction parallel to their environment as determined by traction force microscopy or micropillar deflection techniques. In contrast, motile immune cells produce protrusive forces into their environment and, in their case, mechanical balance is achieved at the level of each podosome and not at the cell scale.^93^ More specifically, protrusive forces generated in the F-actin core are balanced by pulling forces produced in the podosomal ring structure,^100^ making these structures autonomous force generators.^93,101^ Protrusion force microscopy experiments demonstrate that podosomes produce forces in the 10 nN range,^101–103^ which are much larger than the pN forces generated by individual actin fibers.^93^ Along with force generation capabilities, recent studies have shown that podosomes are mechanosensitive to topography by aligning to 3D micropattern edges^104^ and to stiffness by generating larger protrusive forces.^105,106^ Combined, these findings demonstrate that these organelles are capable of force generation and sensing to a similar extent as focal adhesions but are also quite different, suggesting that mechanical adhesion-based immune events cannot simply be explained using well-established focal adhesion-based mechanobiological insight.

### Podosome adhesion and matrix degradation functions

In contrast to focal adhesions, podosomes are also recognized as sites of matrix degradation and have important matrix lytic capabilities.^105,107^ Even though podosomes utilize proteinase-rich vesicles to secrete enzymes for matrix degradation,^107^ this function does not appear to affect adhesion. In fact, podosome formation actually appears to be independent of the physicochemical properties of the substrate and the type of ECM coating, which is contrary to focal adhesions.^104^ This mechanism, distinct from focal adhesion formation, allows macrophages the ability to attach to a variety of different surfaces, ranging from hydrophobic metals,^108^ untreated synthetic hard polymers,^109,110^ ECM-uncoated engineered hydrogels^111,112^ to inert agarose^113,114^ as well as to various natural soft gels.^115–117^

While podosomes seem to form on practically any surface, whether the physicochemical nature of their substrate has an effect on macrophage behavior via podosomal activity remains an open question. These structures display a vast repertoire of various integrin subunits (e.g., β1, β2, and β3 for various ECM proteins and chitosan) and other matrix ligand receptors (e.g., CD44 for hyaluronic acid),^111,118,119^ suggesting an important role for cell–matrix interactions. Functionally, macrophages generally prefer substrates with available integrin binding sites^112,120–122^ and not only tend to spread equally well on ECM-coated substrates^123^ but also seem to better attach to and spread on bare glass, polyurethane, and dextran over chitosan and hyaluronic acid.^124–126^

### Emerging physical models for innate immune cells

In contrast to adherent tissue cells, macrophages do not seem to have a global mechanically balanced “pulling” cytoskeleton, but instead appear to be more plastically deformable with a cortical cytoskeleton that stabilizes their plasma membrane and relies on local “pushing” cell–matrix attachments, which exhibit poor global interconnectivity. These podosomes not only allow macrophages to generate forces and respond to their mechanical environment but also allow them to degrade their surroundings and sample its physicochemical properties. A cell–matrix feedback mechanism that captures these fundamental podosome features has been proposed^92^ and recently supported:^105^ podosomes cluster together, generate smaller protrusive forces, and decrease their degradative capability on soft substrates, but instead individually and forcefully probe their environment while their degradative function is enhanced on stiff substrates.^105^ On the other hand, the mechanical nature of neutrophils and monocytes appears to be less influenced by attachment to their surroundings^97^ and seems to be mostly determined by their global mechanical state.^73^

While these studies into the physical nature of innate immune cells provide us with fundamental knowledge, these descriptions of cellular physicochemical mechanisms of the innate immune system are a critically important guide in understanding the contribution of innate immunity to disease as well as immune responses to biomaterial implantation. To better bridge this foundational biophysical understanding of the innate immune system with practical therapeutic applications, various engineering tools^127–130^ can be utilized, to further develop this understanding, and also to (i) measure the biomechanics of innate immune cells to generate integrative functional metrics of the immune cell phenotype and (ii) build more realistic *in vitro* innate immune microenvironments that better capture those present in the body. A biomechanical understanding of innate immune cells can help us generate mechanical biomarkers to track immune activity, whereas more faithful engineered platforms can be used for drug discovery and mechanobiological insights, obtained from these advanced *in vitro* models, can guide immuno-informed tissue engineering strategies. Here, we highlight engineering approaches to explore the biophysical nature of the innate immune system in the context of cell migration, phagocytosis and mechanobiology.

## UNDERSTANDING INNATE IMMUNE CELL MIGRATION IN ENGINEERED MICROENVIRONMENTS

### Microfluidics and micropillar arrays to visualize immune vascular migration events

**FIG. 3.**
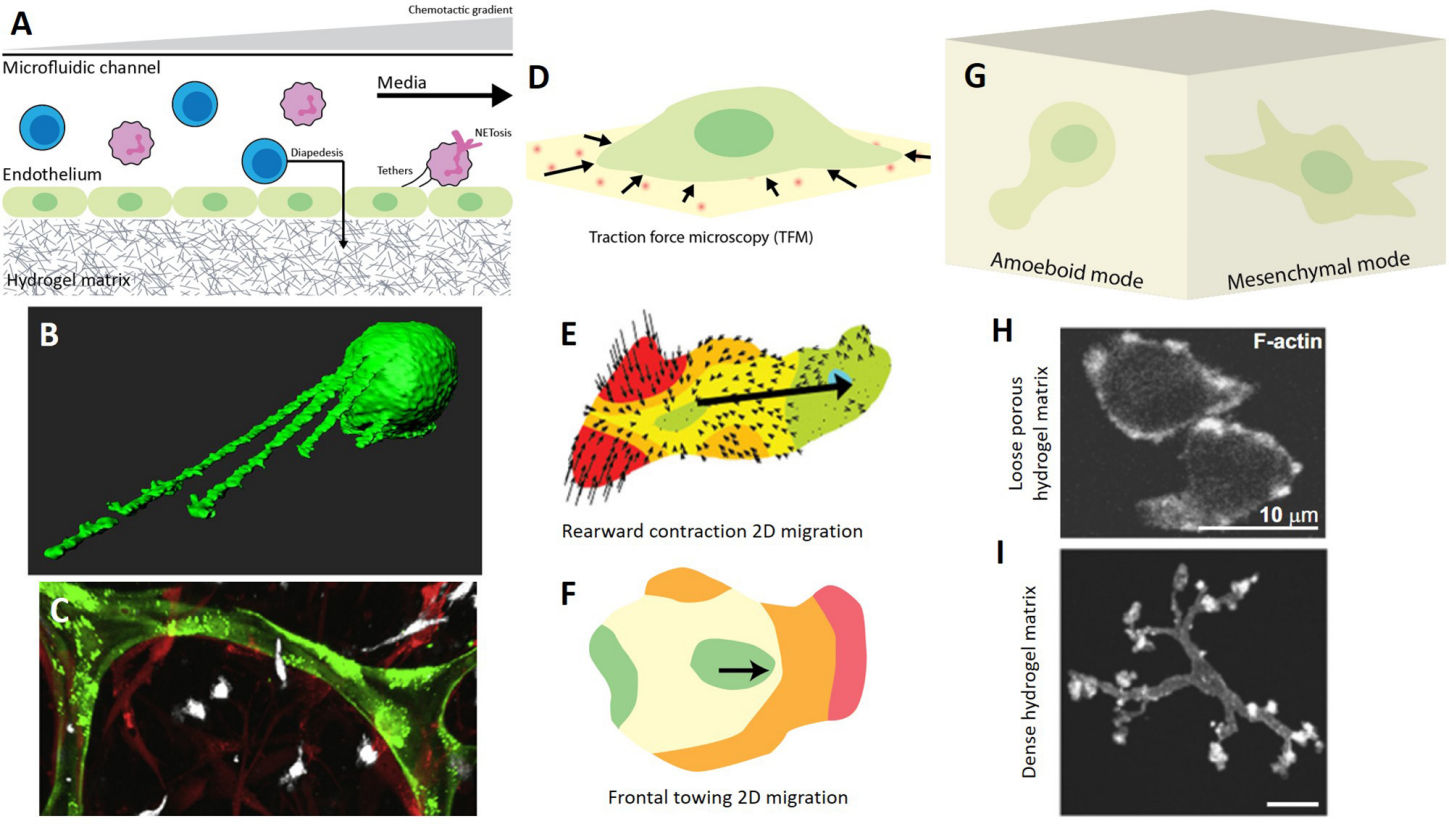
Innate immune cell migration. (a)–(c) Vascular migration events captured using (a) microfluidic technology: (b) neutrophil tethering [adapted from Marki *et al.*, Sci. Rep. **6**, 28870 (2016).^151^ Cpyright 2016 Authors, licensed under a Creative Commons Attribution (CC BY) license] and (c) monocyte (white) transmigration through engineered capillaries (green) into a surrounding fibroblast-rich fibrin matrix [reprinted with permission from Boussommier-Calleja *et al.*, Biomaterials **198**, 180–193 (2019). Copyright 2019 Elsevier.^156^]. (d)–(f) 2D migration on surfaces studied with (d) traction force microscopy where displacements of fluorescent fiducial markers are used to calculated cell traction forces: (e) ameboid rearward uropod contraction migration mechanism displayed by a neutrophil [reprinted with permission from Jannat *et al.*, Biophys. J. **101**, 575–584 (2011). Copyright 2011 Elsevier^166^] and (f) macrophage exhibiting the mesenchymal frontal towing migration mechanism. (g)–(i) Macrophage migration within interstitial tissue mimics: (h) ameboid migration in a fibrillar collagen matrix and (i) mesenchymal migration in a dense collagen hydrogel matrix (h) and (I) reprinted with permission from Maridonneau-Parini *et al.*, Immunol. Rev. **262**, 216–231 (2014). Copyright 2014 John Wiley and Sons.^177^

Although the ability of microfluidic systems to precisely manipulate tiny fluid volumes has been successfully leveraged and combined with “omics” biology to create powerful diagnostic technologies through innate immune cell cytometry and secretome analysis, microfluidics can also be used to simulate the vascular environment *in vitro* and enable the visualization of key immune vascular migration events^131–134^ [Fig. 3(a), Table I]. More specifically, this technology has been extensively used to study neutrophil chemotaxis in response to spatially- and temporally defined^135–137^ soluble chemotactic gradients and has yielded important insights into neutrophil biology. When seeded into singular chemotactic gradient microfluidic devices, neutrophils migrate up the gradient until they reach the maximal concentration,^138^ and only stop to repolarize and migrate along a new gradient.^139^ Their ability to follow these chemical signals is quite efficient, as they robustly pick the shortest route to their destination in maze-like microfluidic circuits.^140^ In the presence of multiple chemoattractant gradients, neutrophils begin to migrate in an oscillatory pattern, sequentially locking in on each source.^141^ However, when subjected to spatially opposing sources, neutrophils integrate these chemical signals and, although they often begin migrating faster toward the stronger more dominant source, the combined presence of both can act synergistically to potentiate neutrophils to also migrate in the opposing direction toward less dominant sources.^142^

**TABLE I.** Engineered platforms for innate immune cell migration.

Platform	Advantages	Disadvantages	Specific applications
Boyden chamber assays	• Numerous commercial products available • Multiwell plate integration and high throughput	• Poorly defined chemotactic gradients • Live cell imaging incompatibility • Resolution limited to cell populations	Transmigration^153^
Microfluidics	• Some commercial products available • Low requirements for cell number and reagent volume • Well-defined chemotactic gradients • Live cell imaging	• Poor reproducibility and low throughput • Technical expertise and troubleshooting is sometimes required	Chemotaxis,^135–142^ neutrophil swarming,^143^ NETosis,^144–146^ margination,^147^ adhesion,^148–151^ and transmigration^152,154–156^
Silicone micropillar arrays	• Live cell imaging • Force quantification	• Technical expertise and troubleshooting is necessary • Poor reproducibility and low throughput	Transmigration^157,158^
Synthetic hydrogels (e.g., polyacrylamide)	• Control over substrate physical characteristics • Force quantification	• Limited physiological relevance	Transmigration^159,160^ and 2D crawling^163–170^
Natural matrices (e.g., collagen and Matrigel)	• *In vivo*-like physiologically relevant microenvironment • 3D cell culture	• Poor control over microenvironmental physical characteristics	Interstitial 3D migration^97,173–176^

Aside from chemotaxis, microfluidics have also been implemented to observe neutrophil swarming^143^ and NET generation (NETosis).^144–146^ In the former application, a microscale array of zymosan particle clusters can be made for neutrophils to interact with. Neutrophils initially randomly encounter these clusters, but – after a few initial interactions and in the span of an hour – begin recruiting other mobile neutrophils to preferentially form swarms around larger clusters, akin to sealing sites of infection *in vivo*. This characteristic behavior is reproducible across different healthy donors and, quite interestingly, is not observed with neutrophils obtained from trauma and sepsis patients.^143^ Separately, microfluidics applied to study NETosis have demonstrated that NETs act as permeable obstacles that separate red blood cells (RBCs) from plasma and, when trapped in capillary microfluidic networks, mechanically perturb blood flow, creating downstream voids of red blood cell traffic. This mechanical vascular process, distinct from coagulation and thrombosis, could be responsible for tissue hypoxia and secondary organ injury during severe inflammation.^144^

Alongside these studies, microfluidic systems have also been employed to more realistically recreate some of the earlier innate immune cell migration events: margination, adhesion, and transmigration. By flowing red blood cells (RBCs) with peripheral blood mononuclear cells (PBMCs) in a variety of microfluidic channels, margination can be readily observed, especially at lower fluid shear rates and downstream of sudden capillary expansion,^147^ supporting the observation that leukocyte adhesion occurs preferentially in postcapillary venules. Adhesion to the vascular endothelium has been simulated on microfluidic surfaces functionalized with selectin molecules. Quite interestingly, neutrophils have been observed to extrude long thin membrane tethers from their microvilli to stabilize themselves on these surfaces, allowing them to adhere under a broad range of shear stress conditions^148,149^ [Fig. 3(b)]. Furthermore, tethers produced at the rear of neutrophils do not retract and, in about 15% of cases, wrap around the rolling cell body to be then “thrown” forward as slings to attach to the surface upon contact, laying out adhesive trails in front of migrating neutrophils. These cell-generated structures not only facilitate neutrophil rolling but also allow the neutrophils to propel themselves forward as tether breakage causes neutrophils to microjump forward.^150,151^ To observe transmigration or diapedesis, microfluidic devices can be designed to have two compartments separated by a thin porous synthetic membrane^152^ in a manner analogous to a classical Boyden chamber assay^153^ or a natural collagen scaffold,^154,155^ either of which can be lined with endothelial cells to simulate the vascular barrier. To better replicate an *in vivo*-like capillary architecture, fibrin can be gelled with endothelial cells and fibroblasts to self-assemble into endothelial capillary networks through which monocytes can circulate and extravasate through [Fig. 3(c)]. In this system, after actomyosin-based transmigration, inflammatory monocytes slow down and become more macrophage-like,^156^ mimicking differentiation that occurs *in vivo*.

Microfabrication-based approaches can also be utilized to produce silicone micropillar arrays, which can be applied to both visualize transmigration and also measure associated mechanical forces. Neutrophil and monocyte penetration through the TNFα-activated endothelium suspended atop of these arrays results in a minute-long force spike caused by outward endothelial cell displacements, which are recovered when the endothelium reseals itself after the process is complete.^157,158^ Alternatively, synthetic polyacrylamide hydrogel substrates seeded with endothelial cells can also be used as an *in vitro* transmigration assay. Although cells cannot actually penetrate unmodified polyacrylamide, neutrophils do go under the endothelium by creating gaps in it, which facilitates the transmigration of subsequent neutrophils.^159^ Although micropillar experiments suggest that the endothelium is most likely responsible for these forces – as functionalized beads also reproduce this effect,^158^ both HL60 neutrophils and endothelial cells in the polyacrylamide system separately generate contractile forces that perturb cell–cell junctions during transmigration. In particular, neutrophils actively appear to push themselves through the endothelium, allowing them to penetrate the endothelium much faster than functionalized beads.^160^

### Synthetic hydrogel surfaces to study 2d immune migration

Hydrogel biomaterials are commonly used in cell culture as replacements for plastic or glass dishes, which poorly replicate the structural and mechanical aspects of the *in vivo* microenvironment.^161^ Synthetic polyacrylamide hydrogels, in particular, have been implemented for studies of innate immune cell 2D migration, which can be observed during vascular crawling and on artificial implant surfaces. Similar to micropillar deflection force measurements, fluorescent tracking beads can be embedded in polyacrylamide hydrogels to measure cell traction forces [Fig. 3(d), Table I]. In addition, polyacrylamide hydrogels can be functionalized with a variety of ECM molecules and hydrogel elasticity can easily be tuned by cross-linking, allowing investigations into the combined physicochemical effect of both ECM coating and stiffness.

Although neutrophils are generally considered to live in suspension, they adhere to fibronectin-coated surfaces by first shedding their microvilli, initiating intimate contact at one point on the surface and then spreading quickly to form another spot of intimate contact.^88^ These two polarized adhesive spots become the lamellipod and the uropod, which are the two characteristic ameboid structures observed in spread and migrating neutrophils.^162^ As observed on micropillar array substrates, neutrophils produce propagating outward forces during initial stages of spreading and then generate steady-state inward peripheral contractile stresses when well-spread. Spreading dynamics appear to be primarily affected by actin cortical shell dynamics, while long-term contraction is mediated by myosin.^89^ When seeded on polyacrylamide traction force-measuring substrates, neutrophils adopt the same morphology and, in contrast to adherent tissue cells, generate contractile forces in the migration direction using their uropod at the back of the cell [Fig. 3(e)], especially when stimulated with chemokines.^163–166^ The direction of uropod traction stresses seems to precede turns during migration, which suggest that uropod contraction might be responsible for controlling directionality.^163^ The generalized biophysical model for neutrophil migration appears to be described by myosin-induced rearward contraction,^166^ which allows neutrophil movement through the rupture of adhesive contacts. It is unclear how stiffness mechanistically affects their migration, but it is clear that neutrophils spread more and display less random more persistent directional migration on stiffer substrates.^164,165^

Macrophages, in contrast, can migrate using both ameboid and mesenchymal modes on 2D surfaces. THP-1 macrophages seeded on collagen-coated polyacrylamide hydrogels migrate slowly in a podosome-dependent manner on substrates with stiffness approaching that of cartilage and bone but do so rapidly on softer substrates using actomyosin contractility.^167^ Observations of ameboid-like macrophage migration are consistent with traction force experiments conducted on similarly soft fibronectin-coated polyacrylamide hydrogels. PBMC-derived primary macrophages appear to use an adhesion-dependent frontal towing mechanism where they extend pseudopods at their leading edge in order to attach and pull themselves forward, as highlighted by strong myosin-driven leading edge contractile forces^168^ [Fig. 3(f)]. However, mouse bone marrow-derived macrophages (mBMDMs) seeded on integrin-agnostic poly-D-lysine polyacrylamide substrates only migrate slower on stiffer substrates when activated with LPS.^169^ Furthermore, macrophages polarized with inflammatory stimuli appear to become less motile compared to alternatively activated macrophages on integrin-associated collagen and fibronectin polyacrylamide substrates,^167,170^ which is consistent with migration studies on conventional plastic and glass cell culture dishes.^97,171,172^ Taken together, these few studies suggest that macrophage migration is inherently complex and can be modulated by their activation state, substrate stiffness, and the availability of integrin binding sites through surface functionalization.

### Natural 3d ecm-based matrices to simulate innate immune migration in interstitial tissues

Although synthetic hydrogels can be precisely fabricated and functionalized with specific structural, mechanical, and cell attachment considerations in mind, they still lack some of the physical characteristics of the native microenvironment and, more importantly, are not as compatible with 3D cell culture^161^ as natural collagen or Matrigel hydrogel matrices are.

Neutrophils and macrophages cultured in both of these matrices exhibit contrasting migration capabilities [Fig. 3(g), Table I]. More specifically, neutrophils and monocytes can only migrate in porous fibrillar collagen hydrogels [Fig. 3(h)], whereas macrophages can also slowly infiltrate dense matrigel or gelled collagen matrices [Fig. 3(i)] by exploiting their access to the mesenchymal migration mode.^97^ In dense hydrogels, macrophages display a retractable tail and extend numerous leading pseudopodia, which have 3D podosome-like structures at their tips. These podosome rosette assemblies allow macrophages to locally degrade the dense matrix and dig a tunnel in order to migrate through it.^97,173,174^ Macrophages appear to either move in a slow saltatory pattern with sequential degradation and movement steps or in a slightly faster back-and-forth fashion within existing tunnels.^174^ In addition, mesenchymal migration appears to be myosin-independent, whereas ameboid migration is, suggesting that macrophage utilize actomyosin contractility to propel themselves faster in looser matrices. The choice of migration mode seems to depend less on matrix stiffness, but more on porosity.^174^ Further investigations demonstrate that myosin inhibition promotes mesenchymal migration,^175^ which leads to speculation that macrophages default to the ameboid mode until it is inhibited by physical and chemical aspects of their local environment. In terms of macrophage activation, unstimulated M0 and polarized M2 macrophages form podosome rosettes and are able to migrate in denser matrices, whereas M1 macrophages appear motionless.^97^ Similarly, M2 macrophages migrate within fibrin gels toward a chemotactic source, whereas M1 macrophages do so very poorly.^176^ M1 macrophages express more α_D_β_2_ integrins and have stronger attachment to fibrinogen, while M2 macrophages exhibit an intermediate level of integrin expression and are less adhesive.^176^ Taken together, these observations suggest that only certain macrophage populations are able to utilize the mesenchymal migration mode, which appears to be correlated with podosome rosettes and macrophage adhesiveness and seems to echo the more mobile M2 macrophages in 2D settings. Perhaps patrolling macrophages sense and feel whether their physicochemical surroundings appear abnormal to decide whether they need to stay and engage or to migrate away and keep patrolling. Furthermore, it seems like inflammatory macrophages opt for the former options, whereas alternatively activated macrophages for the latter.

## MEASURING INNATE IMMUNE CELL BIOMECHANICS DURING PHAGOCYTOSIS

### Pseudopodia dynamics prior to phagocytosis

The mechanical event, typically preceding phagocytosis, is the physical act of finding a target, attaching to it and then pulling it toward the cell body for engulfment and ingestion to occur. Innate immune cells are inherently efficient at doing so and, as a result, recreating phagocytosis *in vitro* is not particularly difficult and can be done with a variety of targets, ranging from living microorganisms to seemingly inert synthetic particles.^178,179^ Notable experiments with immunoglobulin (IgG) functionalized beads have shown that macrophages contact their targets by extending actin-rich filopodium and membrane ruffle structures.^180^ Filopodia extend up to 8 *μ*m away, allowing macrophages to scan and actively probe their surroundings for up to 2 min.^181,182^ Furthermore, studies with surface-bound bacteria have demonstrated that macrophages use a hook-and-shovel mechanism to bind onto them long enough to sever bacterium-surface attachments by inducing the local protrusion of a lamellipodium under them.^182^

**FIG. 4.**
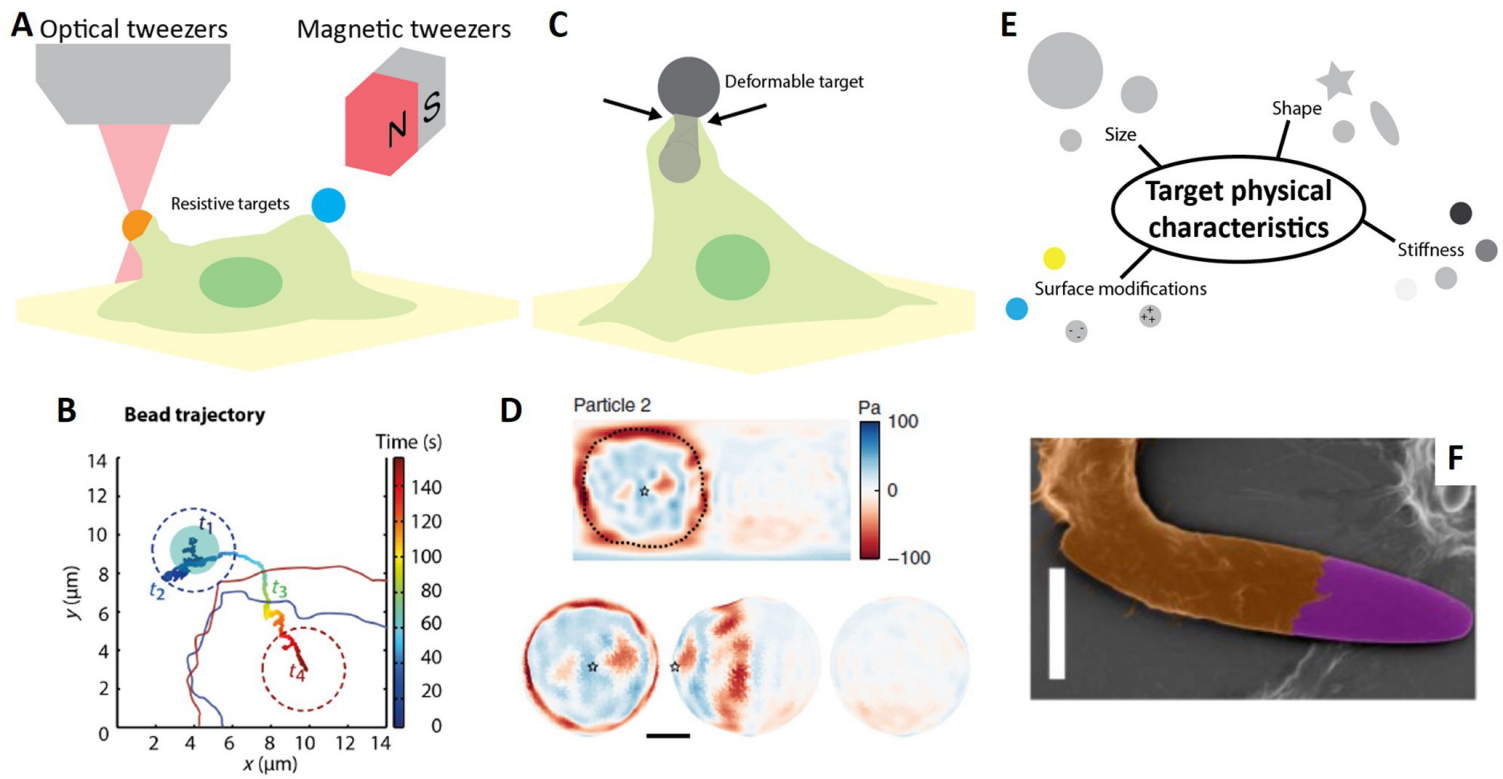
Mechanics of phagocytosis. (a) Cell biomechanics techniques adapted to study pseudopodia dynamics prior to phagocytosis. (b) Bead trajectory following macrophage target approach and uptake. [reprinted with permission from Schuerle *et al.*, Sci. Robot. **2**, aah6094 (2017).^183^ Copyright 2017 AAAS] (c) and (d) Deformable target-based three-dimensional traction force microscopy with (d) a characteristic normal traction force profile for a partially engulfed target [adapted from Vorselen *et al.*, Nat. Commun. **11**, 1–14 (2020).^198^ Copyright 2020, Authors licensed under a Creative Commons Attribution (CC BY) license]. (e) Target physical characteristics that affect phagocytosis dynamics. (f) Scanning electron micrograph of a macrophage ingesting an opsonized elliptical disk polystyrene particle [10 *μ*m scale bar; adapted and reprinted with permission from Champion *et al.*, J. Controlled Release, **121**, 3–9. Copyright 2007 Elsevier.^222^]

Existing technologies used to measure cell mechanical properties can be harnessed to create phagocytosis targets whose dynamics can be measured and externally controlled^181,183–186^ [Fig. 4(a), Table II]. More specifically, spherical probes used in magnetic tweezers systems can be used to mimic free-floating and resistive adherent prey.^183^ After identifying one of these passive targets, macrophages appear to first rapidly push it with pN forces, displacing it sideways by a micrometer, before slowly pulling it toward themselves along a C-shape trajectory^183^ [Fig. 4(b)]. When confronted with a resistive target, macrophages are not always successfully able to pull it toward them and instead appear to desperately keep producing larger pushing forces presumably in an attempt to ensure sufficient physical contact by engaging more stable receptor–ligand bonding.^183^ Counteracting forces, precisely controlled using optical tweezers, also affect pulling dynamics by decreasing filopodia retraction velocities from 300 to 600 nm/s at <1 pN down to 40 nm/s for forces above 15 pN,^184^ while larger forces above 0.5 nN completely prevent filopodia retraction.^185^ Nevertheless, efficient filopodia retraction proceeds in a 36 nm stepwise actin-dependent manner^184^ and occurs in three phases: an initially slow phase, followed by rapid retraction with an average velocity of 85 nm/s and then culminating with slow 10 nm/s uptake. Specific binding appears to be necessary for rapid retraction since targets functionalized with nonspecific amine groups are dragged and transported along the cell surface with no clear directed motion inward, suggesting that certain signaling pathways responsible for actin cortex reorganization are not activated.^181^

**TABLE II.** Engineered targets used to study phagocytosis.

Target	Advantages	Disadvantages
Biological prey (e.g., bacteria and red blood cells)^182,187,188,211,212,218^	• Physiologically relevant targets	• Poor control over target physical characteristics • Inability to quantify forces
Free-floating synthetic microparticles^180,205–210,213–216,219^	• Control over target physical characteristics	• Limited physiological relevance
Resistive probes (e.g., magnetic tweezers and AFM)^181,183–185,196,197^	• External control over target dynamics • Force quantification	• Limited physiological relevance • Poor control over target physical characteristics • Technical expertise is necessary
Flat hydrogel substrates (e.g., polyacrylamide)^192–195,199^	• Some control over target physical characteristics • Some force quantification	• Severely limited physiological relevance
Deformable traction force microscopy hydrogel microparticles^198^	• Extensive ability to quantify 3D phagocytic forces	• Limited physiological relevance • Poor control over target physical characteristics • Technical expertise is necessary

### Mechanics of phagocytosis

Once the prey is sufficiently close, phagocytes can begin to deform their plasma membrane and extend cellular protrusions to encircle the target, forming a phagocytic cup. After the target is fully contained in a phagosome, it is transported deeper inside of the cell where the phagosome fuses with vesicles and lysosomes, allowing the phagocyte to digest and degrade it. Quite early on, researchers noticed that IgG-opsonized erythrocytes are squeezed during the formation and closure of the phagosome, suggesting that mechanical contraction is important.^187,188^ Micropipette aspiration experiments demonstrated that target engulfment and contraction occur sequentially until the phagosome closes^189^ and that membrane tension remains low throughout the process.^190^ Although these studies have provided numerous evidence for mechanics and many of the molecular contractile mechanisms underpinning phagocytosis have already been elucidated,^191^ a more complete mechanical description of phagocytosis has only emerged thanks to the development of specific functional *in vitro* phagocytosis models.

Phagocytes cultured on opsonized substrates perceive it as prey and spread on it in an attempt to engulf it. In this frustrated phagocytosis state, they first rapidly extend pseudopods until membrane tension increases past a threshold level due to the depletion of plasma membrane reservoirs and then they wait for exocytosis to relieve this tension in order to continue.^192,193^ As macrophages spread on traction force frustrated phagocytosis substrates, their actin cytoskeleton first forms into a dense cortical band and then reorganizes into bundles with visible retraction tethers at which point they stop and generate significant contraction forces.^193^ Increasing membrane tension with hypotonic buffers increases phagocytosis efficiency and can even overcome pseudopod extension inhibitors,^192^ while decreasing it with hypertonic buffers improves spreading and shifts the onset of contraction to a later time.^193^ Seeding macrophages on stiffer polyacrylamide substrates encourages them to enter a frustrated phagocytic state^194^ and spread faster, but not further.^195^

Alternatively, magnetic tweezers can be used to quantify changes in the mechanical properties of the phagocytic cup during phagocytosis by actuating the target to rotate in an oscillating magnetic field and measuring the cell's resistance or stiffness to its motion. During phagocytosis, rotational stiffness spikes upwards, but stabilize at a new level, before target internalization is complete, demonstrating the existence of a mechanical bottleneck that must be overcome for phagocytosis to occur. This mechanical bottleneck sets a limit to membrane extrusion and reaches its maximal point when the phagocytic cup extends to the equator of the particle. If the phagocytic cup cannot proceed past it, phagocytosis will stall.^196^ To measure the mechanical forces produced during phagocytosis, macrophages can be confronted with atomic force microscopy (AFM) probes as targets. Macrophages attempting to eat one of these targets first push it with ∼50 pN forces and then, during phagocytosis, pull on it with forces reaching up to 1 nN, as measured from the deflection of the AFM cantilever.^197^ By turning flat polyacrylamide traction substrates into microspheres and quantifying their deformations, three-dimensional phagocytic forces can be non-invasively measured in a realistic setting [Fig. 4(c), Table II]. Initially, macrophages generate not only pushing forces into the target at the initial point of contact but also pulling ones immediately around it. Phagocytic forces then spatiotemporally evolve into a contractile punctate ring that moves along the length of the deformable target progressively squishing it until the phagocytic cup closes [Fig. 4(d)]. In addition, macrophages produce prominent opposing shear forces at the equator of the particle, imposing local torsion on the target.^198^ The punctate character of this evolving contractile belt suggests the involvement of podosomes, especially since podosome-like structures have been recently observed during phagocytosis. These structures are short-lived and expand radially from the site of initial target engagement, allowing the plasma membrane to bend and closely follow the target.^199^ Ultimately, this podosome ring most likely imposes perpendicular forces onto the target, generating a tighter seal and perhaps allowing engulfment through a purse-string mechanism.

### Target physical characteristics

Although phagocytes appear to eat virtually anything, carefully engineered targets can be used to uncover their preferences in terms of target geometry, physicochemical surface characteristics, and bulk mechanical properties [Fig. 4(e), Table II]. Fundamentally, phagocytosis is just an evolved and improved version of receptor-mediated endocytosis and pinocytosis, both of which are common to many other cells. Since macrophages preferentially select phagocytosis to rapidly uptake larger micrometer-sized particles as evidenced by a reduction in clathrin dependence and the formation of actin-rich phagocytic cups,^200,201^ we will focus our attention toward engineering microscale particles, even though numerous studies have tackled the nanoscale.^202–204^

Although there is no particular defining size threshold, maximal uptake of spherical – opsonized or not – polystyrene, polyacrolein, silica, and latex targets by phagocytosis occurs for particles in the size range of 0.5–3 *μ*m,^81,205,206^ which curiously matches with the size of the average bacterium.^207^ Internalization velocity seems to be only slightly affected by target size with larger particles taking more time^205,206^ but is more dominantly affected by target shape with elongated shapes taking much more time.^206,208^ But, more importantly, shape determines whether target uptake is even possible since IgG-functionalized polystyrene worm-like targets^209^ and opsonized cadmium telluride needles^210^ are poorly internalized. In less extreme cases, phagocytosis is possible but requires access to regions of high local curvature, which often correspond to the ends of elongated targets^183,208–211^ [Fig. 4(f)]. In fact, elongated particles^183^ as well as bacillary filamentous bacteria^211,212^ have to be re-oriented and aligned with the cell body long axis for efficient pickup. Not only do bacteria switch from bacillary to filamentous morphology in an attempt to escape phagocytosis by reducing access to its poles,^211^ the imposed formation of a tubular phagosome fails to develop hydrolytic capacity, thereby further improving their survival.^212^ Taken together, phagocytes seem to examine and search their target for a point with high enough local curvature to initiate phagocytosis, which they will successfully complete if target volume does not exceed that of the cell itself.^208^ Along with particle internalization, cell–target adhesion is equally important, as oblate ellipsoids are more efficiently consumed compared to spheres, precisely because their attachment is better.^213^ The prevailing explanation is that maximal cell–target contact engages more receptor–ligand bonding, and thus, results in efficient phagocytosis. Since swollen macrophages lack membrane ruffles and poorly phagocytose microparticles, it has been suggested that these ruffles are responsible in ensuring proper cell–target contact and target geometries have been optimized based on the size of these ruffles.^205,207^

Along with geometrical considerations, certain particle surface characteristics also affect phagocytosis since macrophages preferentially consume hydrophobic^214^ and charged^214,215^ targets. Furthermore, several strategies have been successfully developed to inhibit phagocytosis by coating targets with cell– or protein–repellent polymer coatings.^216^

In terms of target mechanical properties, macrophages show a strong preference to engulf more rigid polyacrylamide (PAA) and polyethylene glycol (PEG) microparticles^194,198,217^ as well as stiffer red blood cells.^195,218^ Quite interestingly, phagocytosis-associated adhesion to stiff targets induces activation of myosin-II that overrides CD47 self-signaling, indicating that mechanical cues can regulate fundamental immunological signaling.^218^ Furthermore, target geometry and mechanics do not act separately, as stiffness becomes especially significant for rod-shape particles.^219^ Bending stiffness has been proposed to unify these target characteristics and is most optimal for phagocytosis when it is neither too low nor too high. Soft nanoconstructs establish short-lived interactions with macrophages, diminishing the likelihood of recognition and internalization, whereas excessively stiff and/or large constructs cannot properly be deformed or internalized even though cell-target adhesion is adequate.^217^

Studying how phagocytosis mechanically occurs and which targets are preferentially ingested by innate immune cells can, not only help us understand how phagocytes physically discriminate between normal and foreign targets but also it can also lead to the design of drug-loaded microparticles that either better evade the innate immune system or specifically target it.^220,221^

## BUILDING PHYSICAL MICROENVIRONMENTS TO STUDY INNATE IMMUNE MECHANOBIOLOGY

**FIG. 5.**
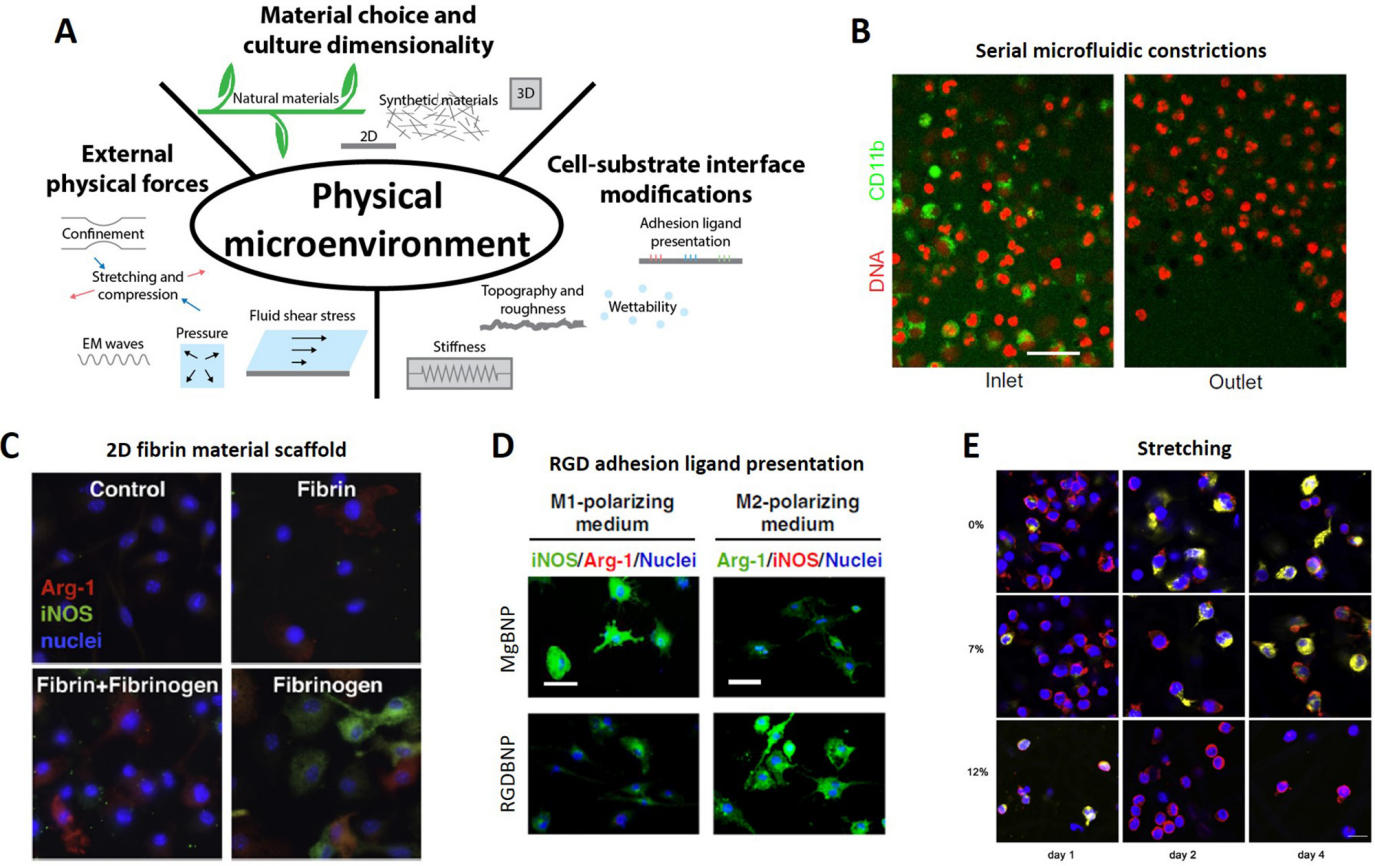
Innate immune mechanobiology. (a) Aspects of the physical microenvironment known to affect innate immune cell behavior. (b) Primed primary human neutrophils depolarize and lose their CD11b surface marker expression after undergoing repeated mechanical deformation in the form of serial microfluidic constrictions [50 *μ*m scale bar; adapted from Ekpenyong *et al.*, Sci. Adv. **3**, e1602536, (2017).^83^ Copyright 2017, Authors licensed under a Creative Commons Attribution (CC BY) license]. (c) Mouse bone marrow-derived macrophages (mBMDMs) exposed to soluble fibrinogen acquire a pro-inflammatory iNOS+ phenotype when grown on tissue culture polystyrene (TCPS), but polarize into an anti-inflammatory Arg-1+ phenotype when cultured on fibrin gels in the presence of fibrinogen [adapted and reprinted with permission from Hsieh *et al.*, Acta Biomater. **47**, 14–24 (2017). Copyright 2017 Elsevier^116^]. (d) RAW 264.7 murine macrophage M2 polarization is enhanced with RGD adhesion ligand presentation, while M1 polarization is supressed [50 *μ*m scale bar; adapted with permission from Kang *et al.*, Nat. Commun. **10**, 1696 (2019).^122^ Copyright 2019, Authors licensed under a Creative Commons Attribution (CC BY) license]. (e) Human peripheral blood mononuclear cells (hPBMCs) grown on poly-ε-caprolactone strips subjected to a 0.8 Hz cyclic uniaxial strain of 7% acquire a CD163+ (yellow) M2 phenotype over time, while those exposed to a 12% strain remain in a CCR7+ (red) M1 state. [Reprinted with permission from Ballotta *et al.*, Biomaterials **35**, 4919–4928 (2014). Copyright 2014 Elsevier.^280^]

As observation of innate immune activity in the body presents considerable challenges, reconstructing the physical elements of the innate immune microenvironment offer considerable opportunities to understand immune-related processes such as neutrophil priming and macrophage phenotype polarization in realistic contexts. Although the biology behind neutrophil activation is not yet fully understood, numerous efforts have already demonstrated that physical cues, such as adhesion^223–226^ and shear stress,^227–229^ modulate activation along with cell deformation^83,230^ [Fig. 5(a), Table III]. In particular, adhesion through various β2 integrin ligands can accelerate neutrophil crawling under high shear stress^224^ and selectin-mediated adhesion can limit the premature activation of neutrophils *in vivo.*^225^ Optically patterned agarose with surface-bound formyl peptides can replicate neutrophil haptotaxis *in vitro.*^231^ Furthermore, shear stress makes neutrophils resistant to priming^227^ but promotes their activation in the presence of platelet-activating factor.^228^ In addition, deformation of neutrophils by optical stretching appears to not only activate them but also inactivate chemically primed ones.^83^ Engineered microfluidic^78,83,232–234^ and sandwich hydrogel^235,236^ environments have been especially useful in probing this physical cue by deforming leukocytes through external confinement. Notably, microfluidic confinement causes neutrophils to replicate *in vivo*-like retrotaxis behavior^233^ and serial constrictions lead to loss of neutrophil activation following chemical priming^83^ [Fig. 5(b)].

**TABLE III.** Simplified macrophage phenotype polarization design considerations for *in vitro* culture models and implants.

Design category	Pro-inflammatory characteristics	Mixed characteristics	Anti-inflammatory characteristics
Material choice and culture dimensionality	• Synthetic materials: poly(ethylene glycol) diacrylate^244^ • Natural materials: chitosan^242^	• Synthetic materials: poly(urethane urea)^241^, polylactic acid^242^ and poly(lactic-co-glycolic acid)^240,243^ • Natural materials: hyaluronic acid^245^	• Synthetic materials: degradable polar hydrophobic ionic polyurethane^239^ and gelatin methacryloyl^244^ • Natural materials: fibrin^116^
Cell-substrate interface modifications	• Adhesion: β2 integrin ligand^272^ and fibrinogen^123,272^ • Wettability: hydrophobic^272^ • Bulk mechanical properties: stiff^167,274,278^	• Adhesion: fibronectin^123,272^ and collagen I^123^ • Roughness^252–256^ and topography^257–267^	• Adhesion: β1 integrin ligand^121,122,244,272^, laminin^123^, matrigel^123^ and vitronectin^123^ • Wettability: hydrophilic^253,270–272^ • Bulk mechanical properties: soft^113,167,274^
External physical forces	• Stretch^280–285^	• Compression^114^ • Hydrostatic pressure^290,292^	• Interstitial fluid shear stress^294^

Even though macrophage mechanobiology has been tackled to a much greater extent, there is still no consensus on how microenvironmental physical cues [Fig. 5(a), Table III] drive macrophage behavior and trial-and-error studies seem to produce contradictory results^39,237,238^ Since this is most likely due to the presence of both biological and physical confounding factors, macrophage mechanobiology should be approached carefully in a more systematic manner. The following subsections briefly describe the subject from this perspective and may have value as a guide in designing both implants and *in vitro* culture models.

### Material choice and culture dimensionality

Compared to anchorage-dependent cells, macrophages appear to be much more sensitive to the material nature of the surfaces they attach to. On degradable polar hydrophobic ionic polyurethane, PBMC-derived macrophages secrete less inflammatory TNFα and more anti-inflammatory IL-10 cytokines,^239,240^ whereas – on poly(lactic-co-glycolic acid) (PLGA) – they secrete more TNFα and IL-10 cytokines, compared to tissue culture polystyrene (TCPS).^240^ Other synthetic materials such as poly(urethane urea) reduce macrophage TNFα secretion, while titanium promotes IL-10 secretion.^241^ Similarly, macrophages grown in synthetic 3D microenvironments such as polylactic acid (PLA) scaffolds secrete more IL-10 compared to TCPS.^242^ Furthermore, 3D nanofibrous PLGA meshes are associated with the secretion of less TNFα when compared to 2D controls.^243^ THP-1 monocytes encapsulated in gelatin methacryloyl (GelMA) hydrogels display an anti-inflammatory regenerative phenotype, whereas those in PEG diacrylate (PEGDA) have an inflammatory phenotype, as explained by the presence of α2β1 integrin ligands in GelMA. The presence of this ligand drives THP-1 monocyte differentiation toward an M2 macrophage phenotype, whereas the absence of this ligand leads to M1 polarization.^244^

In terms of biological materials, when cultured in 3D collagen gels, PBMC-derived macrophages secrete less TNFα and IL-10 in contrast to 2D controls.^245^ Furthermore, inflammatory macrophages secrete more cytokines, whereas M2 macrophages seem to be unaffected by the 3D collagen gel environment.^246^ When macrophages are cultured on 2D hyaluronic acid collagen gels, they release less TNFα and IL-10 compared to unfunctionalized collagen controls; however, this effect is not readily observed in 3D culture where macrophages secrete more TNFα and less IL-10.^245^ Murine model macrophages grown on hyaluronic acid nanofibrous scaffolds express less iNOS (M1) and, with the addition of LPS, proliferate less and secrete more IL-10, when compared to TCPS controls.^126^ Furthermore, their interaction with hyaluronic acid seems to fundamentally negate LPS-induced cell flattening and spreading.^126^ Separately, mouse bone marrow-derived macrophages (BMDMs) exposed to fibrinogen produce TNFα and, when attached to collagen–fibrin gels, predominantly secrete IL-10. However, when the BMDMs seeded on collagen–fibrin gels are stimulated with inflammatory mediators such as fibrinogen, LPS, or IFNγ, they barely release any TNFα, suggesting a fibrin M1 polarization protective effect [Fig. 5(c)]. In addition, these macrophages express less iNOS (M1) and more Arg-1 (M2) genes.^116^ Other materials derived from other natural sources such as chitosan stimulate TNFα secretion by PBMC-derived macrophages^242^ and various chemically modified alginates promote M2 polarization in encapsulated murine RAW macrophages.^247^

### Physical modifications to the cell–substrate interface

In an attempt to tune the biological response of macrophages to a carefully selected material, certain physical modifications to the cell–substrate interface can be made such as introducing adhesion ligands and controlling their spatiotemporal presentation as well as altering substrate roughness, wettability, and stiffness [Fig. 5(a), Table III].

Although known immune cell ligands such as LAIR-1^248^ and self-CD200^249^ can be covalently bound to plastic dishes to reduce TNFα secretion in both unstimulated and inflammatory macrophages, adhesion ligands can also be covalently coupled to various biomaterials in an attempt to control macrophage polarization. On ECM-coated plastic dishes, mBMDMs show differential activation: laminin, matrigel, and vitronectin lead to higher arginase-1 (M2) expression over fibrinogen and collagen I and even more compared to collagen IV and fibronectin.^123^ While Pluronics or Bovine Serum Albumin (BSA) is used to block nonspecific cell attachment, PDMS stamps can be used to micropattern ECM molecules on a selected surface, allowing for spatial control over the presentation of adhesive ligands. When mBMDMs are seeded on thin 20 *μ*m fibronectin-adhesive strips created on plastic, mBMDMs adopt an elongated morphology and appear to polarize toward an M2 phenotype.^250^ Furthermore, cytoskeletal contractility appears to be integral to this phenomenon,^250^ although this effect only occurs when macrophages are cultured on fibronectin or collagen IV.^123^ In support of these observations, the inflammatory response of macrophages is downsized through the epigenetic suppression of late LPS-activated transcriptional programs when they are cultured not only in the spatial confinement on micropatterned fibronectin adhesive strips but also in microporous 3D PDMS scaffolds.^251^ Along with spatial presentation of adhesive ligands, adhesion can be dynamically controlled over time by using magnetic fields to reversibly uncage arginylglycylaspartic acid (RGD) adhesive β1 integrin ligands, thereby allowing murine RAW macrophage attachment and promoting polarization into an M2 phenotype synergistically with chemical stimulation.^121^ Alternatively, magnesium-bisphosphonate metal coordination chemistry can be used to similar ends and again RGD-enabled β1 integrin adhesion promotes M2 polarization^122^ [Fig. 5(d)]. With either method, RGD-mediated polarization seems to involve ROCK signaling, which is associated with cytoskeletal remodeling and cell contractility.^121,122^

Although it is becoming readily apparent that macrophage polarization into an anti-inflammatory phenotype is possible with the presentation of β1 adhesive ligands, the polarizing effect of substrate roughness, wettability, and stiffness is still up for debate. Rough substrates appear to activate macrophages and direct them to adopt mixed phenotypes^252,253^ with inflammatory^254,255^ and/or anti-inflammatory^256^ features. More controlled topographies such as aligned ridges or grooves,^15,257,258^ electrospun fibrous architectures,^259–261^ nanotubes,^262,263^ and various microstructured arrays^258,264–267^ seem to do the same. Some studies have suggested that topography-mediated polarization is scale-dependent with microscale features reducing inflammatory polarization^260,268^ and increasing anti-inflammatory polarization.^257,260^ Efforts are now being directed toward screening thousands of various topographies using “on-chip” technologies. These approaches suggest that primary PBMC-derived macrophage adhesion is maximized on surfaces containing 5–10 *μ*m micropillars, while M1 polarization is driven by larger more disperse surface features and M2 with smaller densely spaced pillar structures.^267^ Since roughness and wettability are intimately linked, several studies have attempted to decouple the two. Rough, but hydrophilic, plastic surfaces act synergistically with chemical stimulation to polarize human PBMC-derived macrophages into respective M1 and M2 phenotypes.^269^ Furthermore, mouse model macrophages grown on carbon nanofibers^270^ or rough titanium^253,271^ hydrophilic surfaces secrete less inflammatory cytokines and seem to adopt an anti-inflammatory phenotype.^253^ However, opposite effects have also been reported where hydrophilic plastic substrates promote M1 polarization.^110^ Nevertheless, irrespective of surface roughness, hydrophilicity does appear to promote the polarization of mouse macrophages into an anti-inflammatory pro-healing phenotype, as demonstrated on micropatterned hydrophilic and hydrophobic strips. Hydrophilic titanium oxide surfaces preferentially adsorb fibronectin, which stimulates macrophage β1 integrin expression and eventually leads to M2 polarization, whereas hydrophobic substrates instead adsorb more fibrinogen, promoting M1 polarization through β2 integrins.^272^

In contrast to topography, macrophages grown on softer substrates generally appear to display less inflammatory phenotypes. THP-1 macrophages secrete less IL-8 on softer (1.4 kPa) RGD-functionalized PAA-PEG interpenetrating network (IPN) hydrogels^112^ and display an M2 phenotype with attenuated secretion of inflammatory cytokines on soft agarose gels (4–100 kPa).^113^ On collagen functionalized PAA gels, higher stiffness (323 kPa) primes differentiated THP-1 cells to an M1 phenotype while softer substrates (11 and 88 kPa) prime them to an M2 phenotype under respective chemical polarization stimuli.^167^ Studies with mouse BMDMs indicate that they secrete less inflammatory cytokines when cultured on softer (130 kPa) RGD-functionalized PEG gels and stimulated with LPS.^273^ Furthermore, macrophages express less pro-inflammatory genes and secrete less inflammatory cytokines on softer (0.3 kPa) laminin and collagen PAA gels, irrespective of chemical M1 stimulation.^274^ However, other studies show that mouse macrophages have an attenuated inflammatory profile on moderately-stiff (20 and 150 over 1 kPa) PAA substrates when stimulated with LPS.^275^ In addition, human THP-1 model macrophages secrete more inflammatory cytokines on soft (13 kPa) gelatin biomaterials but less on stiffer (55 kPa) gelatin substrates, compared to TCPS.^276^ When cultured in 3D collagen gels (27 Pa), PBMC-derived macrophages secrete more IL-10 and less TNFα when the gel is stiffened by 1-ethyl-3-[3-dimethylaminopropyl] carbodiimide (EDC)-cross-linking (57.5 Pa). However, the addition of sulfated and nonsulfated hyaluronic acid inhibits this effect.^245^ Care must be taken when cross-linking collagen gels since the cross-linking agent can itself have an effect on macrophage polarization: EDC promotes both inflammatory and anti-inflammatory mediator secretion under relevant polarization media, while genipin, which in contrast forms part of the crosslinks, attenuates secretion of many cytokines.^117^ Porcine bone-derived particles and ECM gels have different effects on polarization of mouse BMDMs. ECM gels are much softer (5 vs 30 kPa) and lead to lower TNFα secretion and higher IL-10 secretion.^277^ In extremely stiff (MPa range) polycaprolactone (PCL) and Eucommia Ulmoides Gum (EUG) scaffolds, macrophages adopt differing mixed phenotypes.^278^

### External physical forces

After carefully selecting the material and modifying it for macrophage cell culture, external physical forces such as mechanical stretching and compression as well as fluid shear stresses can be introduced into the culture [Fig. 5(a), Table III]. Extensional mechanical stresses applied to PBMCs cultured on cyclically strained scaffolds promote monocyte-to-macrophage differentiation and enhance macrophage ECM remodeling capabilities through both matrix protein deposition and degradation.^279–281^ Furthermore, mechanical strain polarizes macrophages into an overall inflammatory M1 phenotype,^280,281^ although moderate strains of 7% appear to promote M2 polarization^280^ [Fig. 5(e)]. Strain-driven pro-inflammatory polarization is further supported with increased pro-inflammatory cytokine secretion observed in human alveolar macrophages,^282^ rodent lung macrophage cultures,^283,284^ and in human macrophage model cell lines.^285^ This effect acts synergistically with exogenous lipopolysaccharide (LPS) stimulation^282,283,286^ and addition of titanium particles.^287^ Within collagen gels, macrophages appear to mechanically sense displacements in the fibrillar collagen ECM through stretch-activated channels and α2β1 integrins.^288^ Furthermore, magnetically actuated oscillations of RGD β1 integrin ligands can modulate murine RAW macrophage polarization. Low frequency stimulates adhesion and M2 polarization, whereas high frequencies suppress adhesion and promote M1 polarization.^289^ Compared to extensional strain, vertical compression leads to minor increases in TNFα production by human PBMCs^115^ but to drastic increases in pro-inflammatory gene expression and cytokine secretion in THP-1 model human macrophages, regardless of their initial polarization state.^114^

Apart from scaffold-transmitted mechanical stresses, pressurized culture environments promote secretion of inflammatory cytokines by human PBMCs^290^ and uptake of lipoproteins, leading to the formation of foam cells.^291^ THP-1 model macrophages also seem to produce relevant cytokines in response to pressure,^292^ but this effect only seems significant when exogenous cotton particles are introduced into the culture environment.^293^ In terms of fluid shear stress, macrophages cultured in collagen gel microfluidic devices subjected to interstitial-like flow polarize into an M2 phenotype^294^ and appear to rapidly migrate toward regions of higher pressure.^294,295^ Non-physiological physical stimulation in the form of electrical and magnetic fields as well as shock waves also appears to affect macrophage behavior by steering them toward an anti-inflammatory phenotype.^296–298^

## FUTURE PERSPECTIVES

Innate immune cell biophysics has proven to be an interdisciplinary venture, requiring an engineering approach that incorporates immunology. Carefully designed physical microenvironments can be built to study biophysical innate immune processes such as migration, phagocytosis, and phenotype polarization. Although a complete biophysical description of innate immunity is yet to be elucidated, these engineering platforms have helped uncover podosomes, for example, which might speculatively serve as a physical mechanism underlying innate immune system mechanobiology. Not only do podosomes function as mechnosensitive force-generating cell–matrix attachments but they also allow for matrix degradation and physicochemical sampling of their surroundings. As a result, innate immune cells exploit their podosomes to dig tunnels for migration in dense matrices, form contractile apparatuses for phagocytosis, and adapt to their microenvironment by sensing its physicochemical characteristics. Although currently speculative, insight gained from studies examining specific physical innate immune processes could be instrumental in developing a more complete biophysical description of innate immunity.

Despite these discoveries, challenges from both immunological and engineering perspectives still persist. To study innate immunity as a whole, other innate immune cells such as dendritic cells, eosinophils, and basophils, should also be considered along with lymphocytes, which play a key role in regulating the behavior of neutrophils and macrophages *in vivo*. However, even culturing these cells while maintaining *in vivo*-like phenotypes presents considerable challenges and will require further development in media formulations and culture technologies, prior to being able to incorporate them into engineered models of the immune system. In addition, there is growing evidence that non-immune cells, such as fibroblasts, also interact with the innate immune system to orchestrate tissue responses such as tissue repair^19^ and fibrosis.^299^ Integrating other cell types within existing innate immune physical microenvironments could, therefore, be of interest and could yield novel insight about innate immunity but must be done in a way that prevents selection or growth bias based on co-culture media formulations. Furthermore, care must be taken when selecting innate immune cell populations for biophysical studies, as phenotype polarization is still not well-characterized experimentally^64^ and immunological differences exist between species,^300^ patients,^237^ and tissue-resident macrophages.^237^ Similarly, from an engineering perspective, effort must be directed to ensure that innate immune cells seeded within physical microenvironments do not adversely polarize into an undesired phenotype. Given the dynamic nature of the immune system and the surrounding microenvironment, being able to control when and under what conditions immune cells are activated would, therefore, be an important tool in understanding these complex interacting biological systems. Overall, the ability to engineer tissue microenvironments, using various “on-a-chip” strategies recently developed in the field, could have a critical impact in dissecting and understanding these components, shed light on the local cellular-level decision making processes, and how they connect to the global coordinated behavior of immune response.

Although studying the biophysics of innate immunity is not an easy task, the field is ripe with opportunity and the future looks promising. Although there are numerous other biophysical events that characterize innate immunity such as NETosis^301^ and macrophage fusion,^302^ the innate immune biophysics discussed in this review already show important potential application areas. Mechanical characterization of innate immune cells could enable the development of mechanical biomarkers, which could be used to track innate immune activity in health and disease. Insight obtained from biophysical studies of phagocytosis can help to devise new drug delivery strategies that either target or evade the innate immune system. Efforts in understanding innate immune mechanobiology can pave the way for biomaterial-based immunomodulation strategies, which can be implemented to locally minimize inflammation and promote regeneration without causing systemic effects.^303^ Alternatively, mechanical therapies could be envisaged where exposure to an external physical stimulus could be used to polarize innate immune cells into a desirable phenotype.

## Data Availability

Data sharing is not applicable to this article as no new data were created or analyzed in this study.
